# Comparative Assessment of ELISAs Using Recombinant Saposin-Like Protein 2 and recombinant Cathepsin L-1 from *Fasciola hepatica* for the Serodiagnosis of Human Fasciolosis

**DOI:** 10.1371/journal.pntd.0002860

**Published:** 2014-06-12

**Authors:** Bruno Gottstein, Marianne Schneeberger, Ghalia Boubaker, Bernadette Merkle, Cristina Huber, Markus Spiliotis, Norbert Müller, Teresa Garate, Marcus G. Doherr

**Affiliations:** 1 Institute of Parasitology, Vetsuisse Faculty and Faculty of Medicine, University of Bern, Bern, Switzerland; 2 Servicio de Parasitología, Centro Nacional de Microbiología, Instituto de Salud Carlos III, Majadahonda, Madrid, Spain; 3 Veterinary Public Health Institute, Vetsuisse Faculty, University of Bern, Bern, Switzerland; Centers for Disease Control and Prevention, United States of America

## Abstract

Two recombinant *Fasciola hepatica* antigens, saposin-like protein-2 (recSAP2) and cathepsin L-1 (recCL1), were assessed individually and in combination in enzyme-linked immunosorbent assays (ELISA) for the specific serodiagnosis of human fasciolosis in areas of low endemicity as encountered in Central Europe. Antibody detection was conducted using ProteinA/ProteinG (PAG) conjugated to alkaline phosphatase. Test characteristics as well as agreement with results from an ELISA using excretory–secretory products (FhES) from adult stage liver flukes was assessed by receiver operator characteristic (ROC) analysis, specificity, sensitivity, Youdens J and overall accuracy. Cross-reactivity was assessed using three different groups of serum samples from healthy individuals (n = 20), patients with other parasitic infections (n = 87) and patients with malignancies (n = 121). The best combined diagnostic results for recombinant antigens were obtained using the recSAP2-ELISA (87% sensitivity, 99% specificity and 97% overall accuracy) employing the threshold (cut-off) to discriminate between positive and negative reactions that maximized Youdens J. The findings showed that recSAP2-ELISA can be used for the routine serodiagnosis of chronic fasciolosis in clinical laboratories; the use of the PAG-conjugate offers the opportunity to employ, for example, rabbit hyperimmune serum for the standardization of positive controls.

## Author Summary

To improve the serodiagnosis of human fasciolosis caused by *Fasciola hepatica*, we comparatively evaluated the accuracy of two different enzyme-linked immunosorbent assays (ELISAs) based on the use of two published recombinant antigens. The best performance was achieved with the recombinant *F. hepatica* saposin-like protein-2 antigen (recSAP2). Although the *F. hepatica* E/S antigen exhibited a slightly higher diagnostic sensitivity, the higher specificity performance of recSAP2 renders this antigen very suitable for application in low endemic areas, especially when coupled to an easy and standardized production facility as compared to the relatively complex production procedure for an E/S antigen. Conclusively, the recSAP2-ELISA can be used as a routine individual serodiagnostic test for human fasciolosis, especially when backed up by a compatible clinical history together with other serodiagnostic technique for other helminth infections of the liver, e.g. alveolar or cystic echinococcosis.

## Introduction

In Central Europe, the most frequently encountered autochthonous helminthic infections that require appropriate immunodiagnostic support include both forms of echinococcosis (*Echinococcus multilocularis* and *Echinococcus granulosus*), toxocarosis (*Toxocara* spp.), trichinellosis (*Trichinella* spp.), ascariosis (*Ascaris lumbricoides*, *A. suum*) and fasciolosis (*Fasciola hepatica*). Other helminthoses are diseases encountered in the context of travel medicine and sojourn in tropical or subtropical areas. Generally, the immunodiagnosis of helminthic infections is challenged particularly by the problem of high serological cross-reactivity when using crude or inadequately purified antigens. Another serodiagnostic problem relates also to cancer patients who raise antibodies against predominantly carbohydrate epitopes that might be common to helminth antigens [1], [2], [3], as exemplified e.g. by cross-reactive anti-P1 antibodies that can be elevated in some cancer patients as well as in echinococcosis and fasciolosis patients [4], [5].

Thus far, immunodiagnostic tools/methods for echinococcosis [6], [7], toxocarosis [8], trichinellosis [9] and ascarosis [10] that achieve measures of specificity and sensitivity permissible for routine use or commercialization have been developed. However, the immunodiagnosis of fasciolosis, in Central European regions of low endemicity, has remained a major challenge, and routine diagnostic laboratories are struggling with the selection of a suitable and reliable test. Nevertheless, recent improvements have been published, mainly by Latin and North American groups on the use of purified antigens, such as Fas2 [11], CL1 [12] or FhSAP2 [13], [14]. To date, these antigens have not yet been (i) validated according to the standard/s required of routine diagnostic laboratories operating under Central European infectiological conditions and ISO 17025 norms, (ii) assessed in relation to specificity (e.g., considering cancer patients) or (iii) directly compared with each other for diagnostic performance.

Based on a review of the literature, we selected two promising but different recombinant *Fasciola* antigens, the *F. hepatica* saposin-like protein-2 antigen (SAP2) [15] and the cathepsin L1 cysteine proteinase (CL1) [16] to establish and subsequently assess an optimized ELISA for the serodiagnosis of human fasciolosis. In this assessment, an emphasis was placed on the immunodiagnostic discrimination from other (hepatic) parasitological problems encountered in Central Europe, such as alveolar echinococcosis, toxocarosis and ascariosis, but also other parasitic diseases acquired during overseas travel. In addition, one of the most frequently encountered differential diagnostic problems in hepatic and other organ disorders are tumors, which even upon use of various imaging procedures, may not be readily discriminated from particular parasitoses. Moreover, sera from cancer patients are also known sometimes to cause serological cross-reactivity, as has been documented, e.g. for echinococcosis serology [1], [2], [3], [17], [18]. Therefore, one of the crucial considerations for the present study was the inclusion of sera from 121 cancer patients that had already been previously investigated for their putative cross- or non-specific reactivity with *Echinococcus* antigens [2], [3].

The working hypothesis of the present study was that, if both recombinant antigens exhibit a similarly high specificity, then their direct combination might yield a higher diagnostic sensitivity than when employed as single antigens. Therefore, we compared the ELISAs using recSAP2, recCL1 and recSAP2 plus recCL1 with the conventional ELISA (ISO-17025) using excretory-secretory products from adult *F. hepatica* (Fh_E/S). In preliminary experiments with the conventional FhES-ELISA, we had shown that a conventionally used anti-huIgG-alkaline phosphatase conjugate exhibited the same diagnostic performance as a ProteinA-ProteinG-AP-conjugate [PAG-AP] (Gottstein et al., unpublished). Based on these findings and the fact that for PAG-AP a positive control serum of animal origin can be used, we elected to conduct the present study using PAG-AP.

## Materials and Methods

### Ethics statement

All serum samples from humans were collected as part of public health and clinical diagnostic activities, were available prior to the commencement of this study and were treated anonymously, Samples from blood donors were obtained under informed written consent and provided by the Swiss Blood Transfusion Center (SRK). This study was approved by the IPA Review Board of the Vetsuisse Faculty of Bern, Switzerland.

### Positive reference serum samples

#### Fasciolosis

From 30 sera from people with fasciolosis were available for testing; 18 samples were from Swiss fasciolosis patients that had been diagnosed in the context of an outbreak in 2009 [19], 5 sera were from patients that had entered routine diagnostic investigations following requests by clinicians, in the context of the routine diagnostic performances at the Institute of Parasitology in Bern (cases matching criteria (ii) described below), and 7 other sera were from Spanish fasciolosis patients infection confirmed by coprological examination. Inclusion criteria were as follows: (i) coprological detection of *F. hepatica* eggs by flotation, using three temporally independent fecal samples per patient (n = 17); or (ii): epidemiological (i.e. living temporally and spatially in the outbreak area) and clinical evidence of fasciolosis (i.e. elevated liver enzymes or cholangitis or cholestatic jaundice; ultrasonographically revealed thickening of the gallbladder or dilatation of the bile ducts; marked peripheral eosinophilia) and, also simultaneously, positive *F. hepatica* serology in a FhES-αhuIgG-AP-ELISA that had been validated for routine diagnosis in our laboratory (ISO-17025) (n = 13).

### Negative reference serum samples

#### Other parasitoses

Sera used for assessing test specificities and cross-reactions due to other parasitic infections were obtained from 87 persons with the following clinically, parasitologically and/or histologically proven infections [numbers of patients investigated]: hepatic alveolar echinococcosis (clinical P2/P4-N1-M0 status) [n = 5]; hepatic cystic echinococcosis (clinical CE1 or CE2 status) [n = 5]; infection with *Schistosoma* spp. [n = 8]; *Taenia solium* neurocysticercosis [n = 7]; *Strongyloides stercoralis* infection [n = 6]; Visceral larva migrans (*Toxocara* spp.) [n = 10]; *Trichinella spiralis* infection [n = 8]; filariosis by *Onchocerca volvulus* [n = 6]; ascariosis (*Ascaris* spp.) [n = 10]; *Entamoeba histolytica* liver abscess [n = 7]; visceral leishmaniasis (*Leishmania infantum*) [n = 7]; malaria (*Plasmodium falciparum*) [n = 8]. All of these sera had been pre-selected based on high antibody reactions against antigens of the respective homologous parasite species as tested by ELISA and/or IFAT.

#### Cancer patients

121 adult patients (mean age 57±13 years of age) admitted to the outpatient clinic of the Institute of Oncology, University Hospital of Bern, between 1995 and 1997, who had donated serum for a previous, ethically approved study on echinococcosis serology [2]. The sera were stored at −80°C until inclusion into the present study. Inclusion criteria for the patients had been: histologically confirmed malignancy, no history or radiological finding suggestive of hepatic parasitoses, and ages ranging between 18 and 85 years. The distribution of the different malignancy types was as follows: neoplasm of the gastrointestinal tract (20 cases), lymphoma (31 cases), breast cancer (19 cases), lung cancer (14 cases), prostate/testicular cancer (8 cases), sarcoma (5 cases), leukemia (3 cases), nasopharyngeal neoplasm (2 cases), histiocytosis (1 case), gynaecological neoplasm (5 cases), melanoma (4 cases), myeloma (4 cases), bladder/kidney neoplasm (2 cases), pancreatic neoplasm (1 case), neoplasm of the central nervous system (CNS) (2 cases).

#### Negative control sera

The sera used for the determination of normal ranges and parameters respective to the different antigens were from 20 healthy Swiss blood donors, matched by age and gender the group of the abovementioned fasciolosis patients.

### *F. hepatica* E/S-antigen

Excretory-secretory products (FhES) from *F. hepatica* were prepared as described elsewhere [20]. Briefly, adult flukes were collected from the bile ducts from sheep livers obtained from a slaughterhouse and were washed several times in 0.01 mol/L phosphate-buffered saline (PBS), pH 7.4 at room temperature. The flukes were incubated under sterile conditions at 37°C for 24 h in serum-free RPMI-1640 medium supplemented with 25 mmol/L HEPES buffer, 7.5% sodium bicarbonate, containing 100 µL penicillin and 100 µg/mL streptomycin. The medium was then sedimented (5,000× g for 10 min at 4°C) to remove any remaining particles. The supernatants were collected and then concentrated using an YM-10 membrane filter system (Amicon Corp., Lexington, MA). Protein concentrations were assessed with a Bradford based protein assay (BioRad Laboratories, Cressier, Switzerland).

### *F. hepatica* recombinant saposin-like protein-2 antigen (recSAP2)

A fresh, morphologically intact and viable adult *F. hepatica* was isolated from an ovine bile duct and immediately put into RNA later (Invitrogen) for storage. Using peqGold RNAPure and peqGOLD OptiPure (both PeqLab), RNA was isolated according to the manufacturer's manual and by using a poly-T primer cDNA was prepared with the Omniscript RT kit (Qiagen). The coding sequence of the saposin-like protein-2 antigen (recSAP2) was amplified by PCR (initial denaturation: 98°C - 3 min, amplification: 25×98°C–20 sec, 58°C–20 sec, 72°C–30 sec, and a final 72°C step for 5 min) using the primers FhSAP-forward (5′-CACCAACCCACTGTTCGTGTTAATG) and FhSAP-reverse (5′-CTAGCACAGCTTGATTAAACG). Primer FhSAP-dw contained a N-terminal CACC stretch needed for the directional in-frame cloning of the amplicon (306 bp) into the Champion pET Directional Topo Expression Kit (Invitrogen). Insertion was verified by sequencing, and clones containing a perfect matching sequence were used for pilot experiments of expression. The clone expressing the highest level of recSAP2 was then used for large scale expression: 10 ml of overnight culture were diluted in 1 l Luria Bertani (LB) medium containing 100 µg/ml ampicillin (Sigma) and shaken at 37°C until the OD_600_ reached 0.5. The protein expression was then induced by adding 1 mg IPTG. After shaking for 3.5 h at 37°C, the cells were pelleted by sedimentation (15 min, 4,000×g) and the recSAP2 was isolated under denaturating conditions using 2 Protino Ni-IDA 1000 packed columns (Machery-Nagel) according to the manufacturer's instructions, with the following exception. After washing, under denaturating conditions, the columns were washed with 10 ml non-denaturating buffer (50 mM NaH_2_PO_4_, 300 mM NaCl), and the recombinant protein was eluted three times with 1 ml non-denaturating elution buffer (50 mM NaH_2_PO_4_, 300 mM NaCl, 250 mM imidazole, pH 8.0). To reach ELISA-stage, the recSAP2 was precipitated with saturated ammonium sulfate solution, and the precipitate dissolved in ELISA coating buffer (100 mM sodium carbonate, pH 9.6). Storage prior to use for ELISA was at −80°C.

The purity and antigenicity of the recSAP2 were assessed by silver-staining of SDS-PAGE gels [21] and Western blot analyses, as described previously for recP29, a recombinant antigen of *E. granulosus* [22].

### Recombinant cathepsin L-1 protein (recCL1) of *F. hepatica*

The complete cDNA sequence encoding *F. hepatica* secreted CL1 was retrieved from GenBank. Forward (5′- GTACCCGACAAAATTGACTGG-3′) and reverse (5′- TCACGGAAATCGTGCCACCAT-3′) primers were designed to amplify the appropriate region of the protein (220 amino acid), without the C-terminal propeptide (55 amino acid). A CACC-tag was added to the 5′ end of the forward primer for further cloning into the Champion pET Directional Expression kit (Invitrogen).

The cDNA encoding the CL1antigen (24.2 kDa) was amplified by PCR of 250 ng of *F. hepatica* cDNA as a template (the same as used to amplify recSAP2), 200 µM dNTPs, 0.5 µM of each forward and reverse primer, in a total volume of 50 µl with 1 U of Phusion High-Fidelity DNA polymerase (New England Biolabs). The amplification was carried out using an initial denaturation of 98°C for 1 min, followed by 25 cycles of denaturation at 98°C for 30 s, annealing at 58°C for 30 s and an extension at 72°C for 30 s. The final polymerization was carried out at 72°C for 5 min. The 658 bp PCR product was purified using High Pure PCR Product Purification Kit (Roche) and then cloned into Champion pET expression vector. Competent *E. coli* (TOP10) cells were transformed using the manufacturer's instructions (Invitrogen). The transformed bacteria were incubated on LB plates containing 100 µg/ml of ampicillin at 37°C overnight, and colonies containing the insert were identified by colony PCR. Five positive clones were grown overnight in LB medium containing ampicillin, and then the plasmids were isolated using a QIAprep Spin Miniprep Kit (Qiagen) according to the manufacturer's protocol. Each vector construct was sequenced to ensure an open reading frame. Recombinant CL-1 was expressed as a fusion protein with His-Tag in *E. coli* BL21 as described above for the recSAP2. RecCL1 from *E. coli* was purified under denaturing conditions (8M Urea) using packed columns (Protino Ni-IDA 150, Macherey & Nagel) according to the instructions of the manufacturer. The eluate was passed through PD10 desalting columns (GE Healthcare) and then was dialyzed against PBS. Purified protein samples were examined in silver-stained SDS-PAGE gels [21] and by Western blot [22].

### ELISA

FhES-, recSAP2- and ecCL1-ELISAs were carried out essentially as described for *Echinococcus* antigens [23]. Briefly, sera were diluted 1∶100 and tested using the following antigens (at optimized coating concentrations): FhES-antigen (10 µg protein per ml); recSAP2-antigen (0.1 µg protein per ml); recCL1-antigen (0.1 µg protein per ml). A fourth ELISA included a double-coating with a mix of 0.1 µg protein of recSAP2 and recCL1 per ml. As a conjugate, an anti-human-IgG-alkaline phosphatase [αhuIgG-AP] conjugate (Sigma; 1∶1'000 dilution) or a ProteinA-ProteinG-AP-conjugate [PAG-AP] (Thermo Scientific no. 32391; 1∶10'000 dilution) was used. The four *Fasciola*-antigen-ELISAs validated here were first calibrated, in order to determine the optimal threshold (cut-off value) for the discrimination between positive and negative findings. The individual cut-off value was thus determined by testing blood donor sera and tumor patients' sera and potentially cross-reactive sera (from patients with other parasitic diseases) as a one group together, thus reaching a representative average number of the “negative samples” encountered in a routine laboratory. Inter-test and intra-test variations in test results were calculated as coefficients of variation for reference negative and positive sera, all tested in triplicate on each test plate; variation of ≤15% was recorded, which is considered acceptable for serodiagnostic assays [2].

### Statistical analyses

For statistical analyses, the samples from the 30 patients with confirmed *F. hepatica* infection represented the positive status (1), whereas the 20 healthy individuals, the 121 cancer patients and the 87 patients infected with potentially cross-reacting or diagnostically relevant other parasitic infections, all represented the negative status (0). The comparative evaluation of the four assays (i.e. FhES-ELISA; recSAP2-ELISA; recCL1-ELISA; recSAP2-recCL1-ELISA) was carried out using this classification. To quantify the linear numerical correlation between the raw data measurements of the three assays, Spearman rank correlation coefficients were derived using all 248 samples. The distribution of OD_405 nm_-values for the samples in the three assays was displayed using dot plots and box plots. In a Receiver-Operator-Characteristic (ROC) analysis, threshold (cut-off) values for the following four conditions were derived according to:

- Cut-off for the maximum specificity (SP) where the sensitivity (SE) was still 100%
- Cut-off for the maximum value for Youdens J = SE + Sp −1
- Cut-off for the maximum overall accuracy = (true positives + true negatives)/n
- Cut-off for the maximum sensitivity at which the specificity was still 100%

In addition, modified two-graph ROC curves were drawn for the different assays, and the areas under the ROC curves (AUC) were statistically compared for significant differences. Descriptive statistics, plots and ROC analyses were done Microsoft Excel 2010 (www.microsoft.com) and the statistical software package NCCS 8 (www.ncss.com).

### Attention to cross-reactivity

The highest potential for a false positive interpretation of test results relates to sera from patients suffering from other parasitic diseases. In Table 1, we present the different parasitic diseases and their rate of cross-reactivity determined in the different ELISA-types, yielding a relative specificity index linked to cross-reactivity. To determine the threshold between positive and negative results, the cut-off value was arbitrarily set at the Youdens J maximum value, calculated as described above (MedCalc software version 12.7.5.0; http://www.medcalc.org).

**Table 1:** Degree of cross-reactivity found with the four antigens assessed by ELISA, and based upon a discrimination between positive versus negative reaction (Youdens J max), yielding thus a relative specificity index with regard to cross-reactive parasitic disease sera.

Parasitic disease	ntot	(FhES-ELISA) npos	(recCL1) npos	(recCL1-SAP2) npos	(recSAP2) npos
neuro-cysticercosis	7	1	1	1	0
cystic echinococosis	5	0	0	0	0
alveolar echinococcosis	5	1	1	1	1
hepatic amoebosis	7	0	0	0	0
visceral leishmaniosis	7	0	3	3	0
malaria (falciparum)	8	0	0	0	0
filariosis/onchocercosis	6	2	0	0	0
strongyloidosis	6	0	0	0	0
ascariosis	10	0	0	0	0
larva migrans (toxocarosis)	10	0	0	0	0
trichinellosis (*T. spiralis*)	8	0	0	0	0
schistosomosis	8	0	0	0	0
***Total***	***87***	***4***	***5***	***5***	***1***
***relative specificity***		***95%***	***94%***	***94%***	***99%***

ntot = total number of sera tested; npos = number of positive sera.

### List accession numbers

GenBank accession number for the used cDNA-SAP2 stretch: AF286903.1.

GenBank accession number for the complete cDNA sequence encoding *F. hepatica* secreted CL1: U62288.2.

## Results

### recSAP2 production and analysis

**Figure 1:**
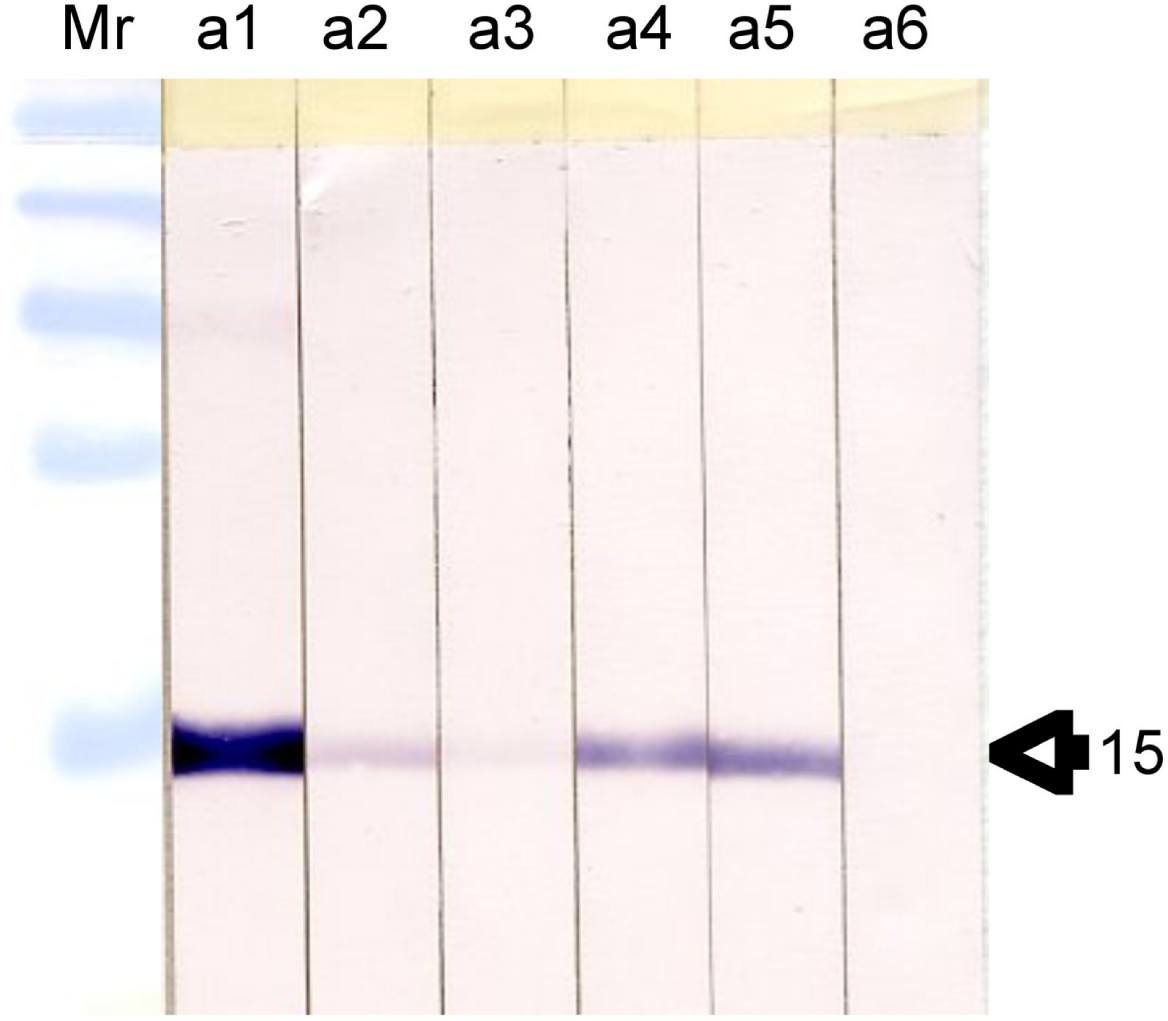
Western blot analyses of recSAP2-antigen. Arrow indicates the 15 Mr size of the revealed recombinant and affinity-purified protein. Western blot findings concerning six different sera from fascioliosis patients with different FhES-ELISA-αhuIgG-AP antibody levels (see text).

Five batches of recSAP2 and five batches of recCL1 were both independently produced and analysed by silver staining (data not shown). As all batches yielded identical purities, they were pooled to obtain two single working batches, respectively. These batches were each assessed with known fasciolosis-sera by Western blot to verify the antigenicity of the two single bands of expected relative mobilities of Mr 15000 and Mr 26000 for recSAP2 and recCL1, respectively (data shown for recSAP2 only, Figure 1). Fasciolosis-sera were selected, such as to cover the whole range of antibody levels measured by the conventional FhES-ELISA. The relationship between “banding intensity” and FhES-ELISA antibody level was relative. Serum a1 has the highest levels in FhES-ELISA (>100 AU, relative antibody units: The quantification of these ELISA-results, expressed in relative antibody units [AU], arises from the routine serology carried out at the Institute of Parasitology in Bern, and is not further specified in this article), and exhibited also the strongest staining intensity by Western blot. Sera nos. a2–a5 yielded medium FhES-ELISA antibody levels (70, 40, 51 and 66 AU), while staining intensity in Western blot varied considerably and appeared not to be directly linked to the recSAP2-ELISA findings. Serum a6 was very weak in FhES-ELISA, and was not detectable by Western blot analysis. However, the same serum (a6) was, nevertheless, also weakly positive in recSAP2-ELISA (Figure 1).

### Distribution of absorbance values (A_405 nm_)

**Figure 2:**
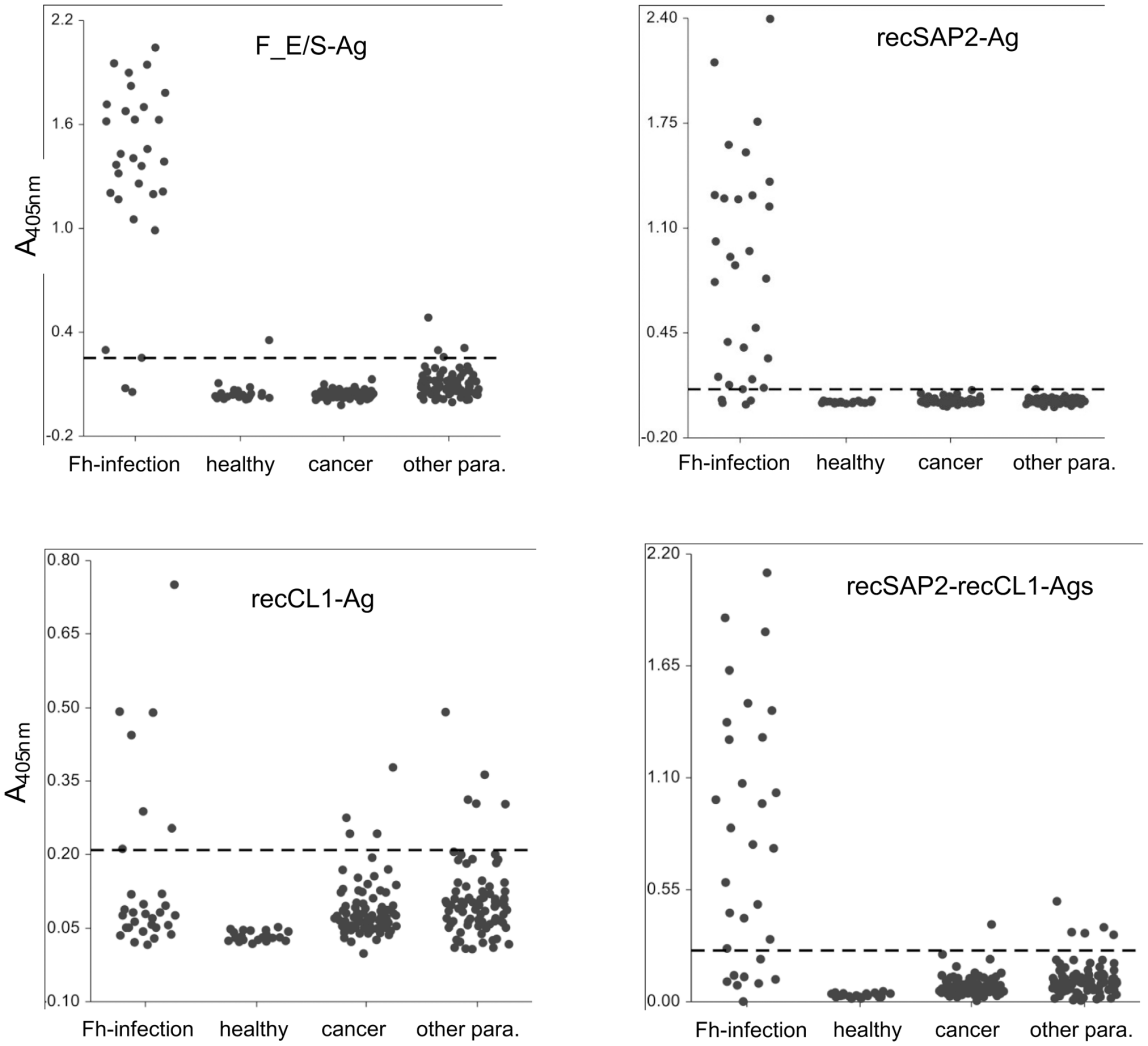
Dot plot showing the distribution of A_405 nm_ values (y-axis) for the four antigens/ELISAs and for the four groups of sera tested (Fh-infection; healthy blood donors; cancer-patients, sera from patients with other parasitoses). The dotted lines indicate the respective Youndens J max values as shown in Tab. 2 and used as threshold discriminating between positive and negative serology.

The distribution of absorbance values varied considerably between test positive and negative samples, as to be expected, but also between different assays (Figure 2), and different optimal cut-off values were established for the four tests (see section of ROC analyses).

### ROC analyses

**Figure 3:**
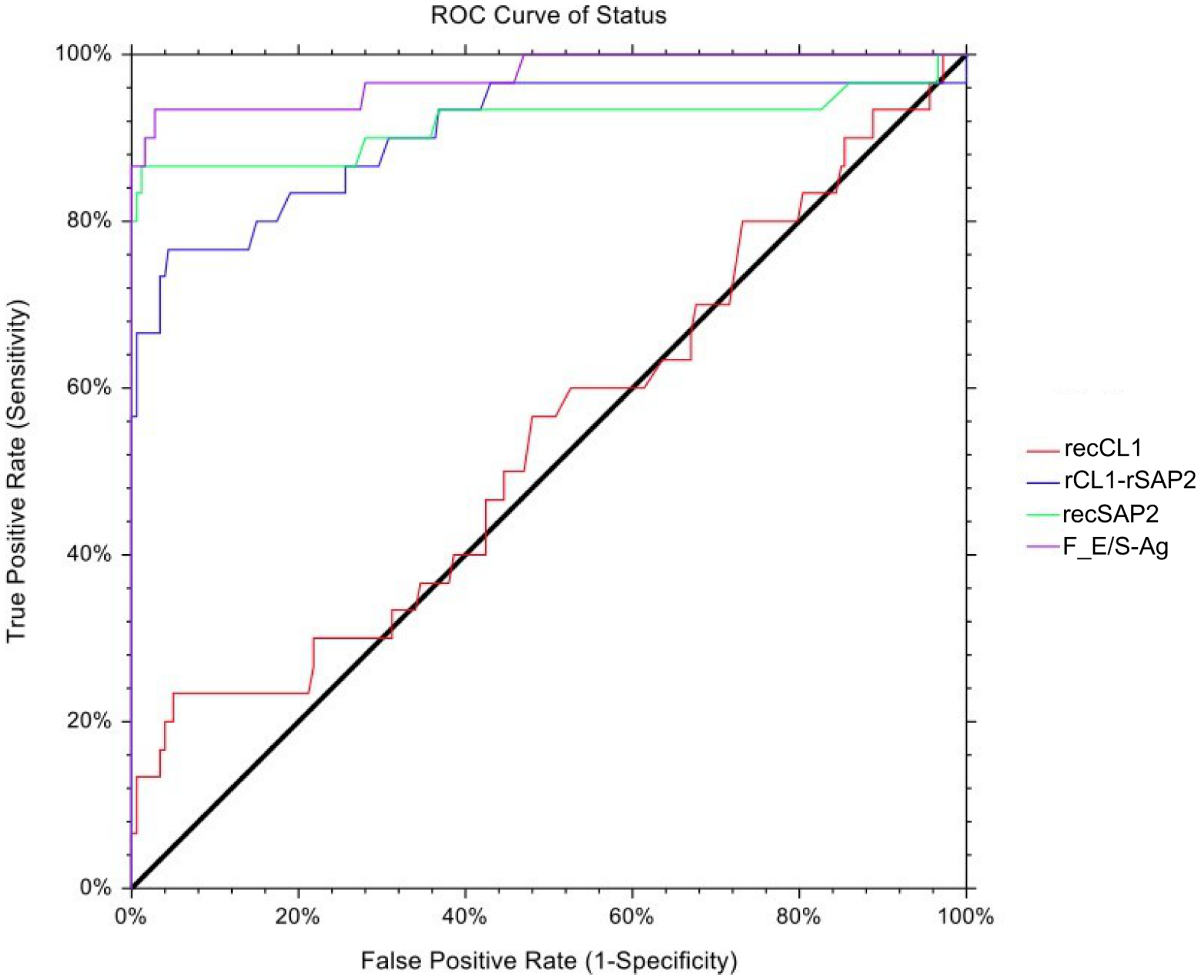
Empirical ROC curves for the four antigens/ELISAs tested.

**Figure 4:**
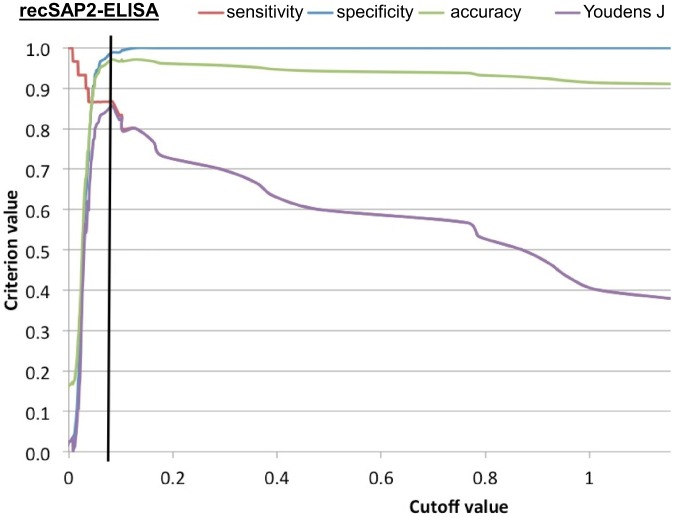
Multi-Graph ROC curve showing the empirical value for sensitivity (red), specificity (blue), overall accuracy (green) and Youdens J (violet) as a function of the cutoff value, exemplified in function of the recSAP2-ELISA selected as best operating ELISA among the four assays investigated. The blue line indicates the cutoff (0.084) at which the overall test performance turned out to be optimal.

The highest overall accuracy (agreement between references status and test result) reached was 0.984, while the highest combined sensitivity and specificity (Youdens J) was 0.905 (Table 2, Figure 3). The recSAP2-ELISA cut-off 0.084 showed the best combination of sensitivity (0.867) and specificity (0.989) of the three recombinant-antigen assays (Table 2). For this assay, the empirical values for sensitivity, specificity, overall accuracy and Youdens J, as a function of the cut-off value, were plotted in a multi-line ROC graph (Figure 4) to illustrate the pattern of these test characteristics over a range of cut-off values. When comparing the AUC values of the four tests, test results of the recCL1 assay were significantly lower than for all other assays (p<0.001), while results of the FhES assay were significantly higher, even when compared with the second best assay (recSAP2; p<0.047).

**Table 2:** Selected cutoff values (*) for which the maximized accuracy criterion (diagnostic sensitivity, diagnostic specificity, overall agreement and combined SE and SP (Youdens J)) was achieved; the table includes also the ROC AUC for the four different *Fasciola*-ELISAs.

Test	Cutoff	Sensitivity	Specificity	Agreement	Youdens J	ROC AUC
recCL1	0.020	**1.000**	0.028	0.167	0.028	0.539
recCL1	0.210	0.233	0.950	0.847	0.183*	
recCL1	0.440	0.133	0.994	**0.871**	0.128	
recCL1	0.490	0.067	**1.000**	0.866	0.067	
rCL1rSAP2	0.000	**1.000**	0.000	0.144	0.000	0.907
rCL1rSAP2	0.210	0.767	0.955	0.928	0.722*	
rCL1rSAP2	0.410	0.667	0.994	**0.947**	0.661	
rCL1rSAP2	0.590	0.567	**1.000**	0.938	0.567	
recSAP2	0.007	**1.000**	0.034	0.172	0.034	0.918
recSAP2	0.084	0.867	0.989	**0.971**	0.856*	
recSAP2	0.084	0.867	0.989	**0.971**	0.856	
recSAP2	0.129	0.800	**1.000**	**0.971**	0.800	
FhES	0.056	**1.000**	0.531	0.598	0.531	0.984
FhES	0.252	0.933	0.972	0.967	0.905*	
FhES	0.989	0.867	**1.000**	**0.981**	0.867	
FhES	0.989	0.867	**1.000**	**0.981**	0.867	

### Cross-reactivity

Cross-reactions, as a results of a positive-negative discrimination based on the cut-off value selected, are presented according to the different parasitic disease groups investigated (Table 1). One serum (a case of alveolar echinococcosis) consistently cross-reacted in all four ELISAs, whereas all other cross-reactions were individually scattered among individual ELISAs. The best score in specificity (99%) was achieved by recSAP2-ELISA, with a single instance of cross-reactivity (described above).

### Diagnostic sensitivity

Using the selected cut-offs, FhES exhibited the best level of diagnostic sensitivity (93%) (28 positive sera of 30 *Fasciola*-cases), followed by recSAP2 (26 of 30 cases; 87%), while the sensitivities achieved using recCL1 and the combination of recCL1 and recSAP2 were all below 77% for all elaborated cut-offs (Table 2).

## Discussion

Coprological diagnosis, based on the identification of *F. hepatica* eggs found in stools, duodenal contents or bile analysis is still commonly employed as a “gold standard” to detect human fasciolosis. This is the case, despite the consensus that this method is not entirely reliable [24] for reasons such as: (i) eggs are not detected until the patent period of infection, when much of the liver damage has already occurred by the migration of juvenile flukes in the liver parenchyma, (ii) eggs are released sporadically from the bile ducts and, hence, stool samples from infected patients may not necessarily contain eggs [25]. Therefore, serological techniques play an important complementary role in the diagnosis of clinical cases of fasciolosis. In the situation of low endemicity, such as encountered in many countries of Central Europe, serological methods require not only a good diagnostic sensitivity, but more importantly also a high specificity. The reason is that potentially cross- or false-positively reacting sera will be much more frequently found in routine diagnosis than actual true cases of fasciolosis. This has to be considered particularly in the context of a differential diagnosis, predominantly related to any other hepatic disorders resembling, symptomatically, those of fasciolosis.

Serodiagnosis of fasciolosis of humans has been successfully performed by employing several antigens (antigen fractions) of *F. hepatica*, where, to date, ES products have become the most commonly used antigen in-house ELISAs [24]. Nevertheless, the use of FhES is associated with several problems when used for routine diagnostic laboratory conditions, including (1) a dependence on the availability of living flukes and (2) representing an antigen mixture that is subjected to variations due to natural and artificial conditions (e.g. time between slaughter and cultivation). This makes antigen standardization between diagnostic laboratories difficult, whereas a recombinant single component antigen exhibits a constant composition. With regard to recombinant antigens, cathepsin L1 (CL1) and saposin-like protein 2 (SAP2) from *F. hepatica* are of the most frequently referenced candidates being used for detecting anti-*Fasciola* antibodies in different epidemiological situations [12], [15], [16], [26], [27]. For the present study, we selected both SAP2 and CL1 as key candidates to be compared, alone or in combination, in the form of bacterially-expressed recombinant antigens (recSAP2 and recCL1). As a serological standard, we used a conventionally employed *F. hepatica* ES antigen (FhES). In order to improve routine applicability of any of these tests, we carried out a preliminary study comparing the efficacy of anti-human-IgG-alkaline phosphatase and a ProteinA-ProteinG-alkaline phosphates conjugate (PAG-AP) to detect anti-*Fasciola*-antibodies (data not shown). We documented a comparable performance of both conjugates (even slightly improved for PAG, although statistically not significant). PAG-AP offers the considerable advantage that it binds also to IgG of several animal species, including, for example, rabbit IgG. The availability of sufficient positive control serum for routine diagnostic application under ISO 17025 accreditation conditions is one of the big problems in routine diagnostic laboratories, as the procurement of such serum (in larger quantities) from fasciolosis patients is difficult or even impossible in countries of low endemicity. Alternatively, hyperimmune serum from rabbits or other appropriately immunized animals can assume the role of positive control reagents, thus considerably facilitating the establishment of standardized operating procedures (SOPs) of *Fasciola*-serology.

The results of the present, comparative study demonstrated similar performances of the FhES and the recSAP2 antigen with regard to diagnostic sensitivity and specificity, whereas the assay using antigen recCL1 did not reach the expected performance. Our initial working hypothesis had been based on the assumption that a combination of recSAP1 and recCL1 would increase diagnostic sensitivity, provided specificity could be maintained. This hypothesis was justified by an advanced appraisal of previous publications on the topic. Carnevale et al. [26], who used a recombinant CL1 containing the proregion of the protein, reported 100% sensitivity and 100% specificity. In this study, however, the selection criteria, from both clinical and epidemiological perspectives, have not been described, such that one cannot elucidate whether 100% sensitivity represents a true diagnostic sensitivity, as encountered in a routine diagnostic laboratory. Similarly, Tantrawatpan et al. [28], who used a peptide-based form of CL1 deduced from *F. gigantica*, reported 100% sensitivity and 99.7% specificity. However, in the latter publication, the pre-selection criteria for the fasciolosis sera were not documented as to allow an assessment of the actual diagnostic sensitivity. Such tests should also include sera from acute cases, for which infection had been proven but eggs could not be detected repeatedly using a coprological sedimentation technique. For example, sera from acute cases were not included in the study by O'Neill et al. [16]; these authors reported 100% sensitivity for an ELISA using CL1 expressed in *Saccharomyces cerevisiae* (yeast) and an anti-IgG4-detection systems following the testing of sera from 26 cases with egg-excretion. However, these authors did not evaluate the true sensitivity by testing sera from cases representing various forms of infection and disease stages. A relatively recent study on recombinant CL1 was carried out by Gonzales Santana et al. [27] upon use of *Pichia pastoris* for expressing recFhCL1. Conversely to our study, the antigen demonstrated not only an excellent diagnostic sensitivity, but also an optimal specificity. The reason for the difference encountered between these study findings and our study may be the found by the fact that expression of a metazoan gene such as *cl1* in *P. pastoris* may much better lead to carboxylation of the antigen, and thus to an improved formation of relevant epitopes within this proteinic antigen. We will address this important feature in our next studies. Another reason why the diagnostic sensitivity of recCL1 was higher in other studies [16] may have been the use of a subclass-specific anti-IgG4-conjugate. The same approach (anti-IgG4-conjugate) was chosen by Tantrawatpan et al. [28], who furthermore employed a peptide-based synthetic FhCL1-antigen. Although we know that the protein A and protein G used in our study, both principally bind to human IgG4 (http://www.amsbio.com/brochures/Protein-A-G%20-Affinity-for-IgG-subclasses.pdf), it will be nevertheless interesting to compare, in future studies, both conjugate types directly with regard to the diagnostic sensitivity yield.

In our study, bacterially expressed recCL1 exhibited two related problems, which finally rendered this antigen not useable for our purpose. First, in comparison to recSAP2, recCL1 displayed relatively high background reactivity with both, sera from cancer patients and sera from patients suffering from other parasitoses (see Figure 2). This translated into a relatively high cut-off level for the discrimination between seropositivity and seronegativity and, thus, resulted in a relatively low diagnostic sensitivity. Nevertheless, we raised the question whether, among the four recSAP2-seronegative fasciolosis patients, one or more of them would be recCL1-positive, thus justifying a possible combination of the two antigens. However, this was not the case. In this context, it is also important to mention that the two FhES-negative fasciolosis patients were also seronegative against the recSAP2 and recCL1 antigens. Consequently, an overall appraisal of the results did not suggest a routine application of recCL1 antigen for the serodiagnosis of human fasciolosis. Nevertheless, as other previous reports clearly documented a good diagnostic performance of recCL1 if produced by a different expression system [16] or as a synthetic polypeptide [28], we will, in future studies, switch from bacterial expression to the other expression/synthesis systems.

A detailed comparison between the diagnostic operating characteristics of the recSAP2-ELISA and the other *Fasciola*-ELISAs included in our study demonstrated clearly an excellent performance of recSAP2 in relation to both specificity and cross-reactivity (specificity 99%, see Tab. 1). Regarding diagnostic sensitivities, among the 30 fasciolosis patients available for our study, only one patient with a coprologically confirmed fasciolosis remained consistently negative in all four tests, including the recSAP2-ELISA. Due to the lack of clinical data from the respective patient, the reason for this false-negative result could not be established. Here, the lack of clinical and radiological evidence might indicate a chronic infection status with a low infection intensity, accompanied by a decline or even disappearance of parasite-specific antibody levels. In this respect, it is possible that especially the hepatic parenchymal migration stage of juvenile flukes early during infection induces the strongest immune response, whereas a few adult worms remaining in the bile ducts during the chronic phase of infection might not be sufficient to sustain an antigen stimulus to maintain a detectable serum antibody level. Overall, sera from four fasciolosis patients were test-negative in both recombinant antigen-based assays, while three of them were clearly seropositive in the conventional FhES-ELISA. However, this slight diagnostic inferiority of recSAP2 as compared with FhES was largely compensated by other parameters that favored SAP2 as a routine serodiagnostic tool, particularly when applied in a low endemicity area. In such a situation, the comparatively higher specificity of recSAP2 (99% *versus* 95%, see Table 1) might be superior to the comparatively higher sensitivity of FhES (87% *versus* 93%). Importantly, in contrast to FhES, recSAP2 did not exhibit occasional cross-reactions with sera from neuro-cysticercosis and filariosis patients (see Table 1). Such cross-reactions might hamper the diagnostic performance in cases where other clinical data are inconclusive, and where serology becomes a crucially important diagnostic tool.

In conclusion, we consider the recSAP2-PAG-AP-ELISA as serological test system for routine diagnosis of human fasciolosis, particularly if test results are supported by clinical history and the use of other serological tests controlling for possible cross-reactions due to antibodies induced by other helminths. In addition, this test system might serve as an excellent serodiagnostic tool for epidemiological studies of human fasciolosis, particularly in the context of outbreaks, or accumulated case numbers, for example, as observed recently in Switzerland [19]. Our conclusions are in perfect agreement with a previous report from Figueroa-Santiago et al. [15], who were the first authors to document the excellent diagnostic performance of the recSAP2-ELISA.
